# Integrating Spatial and Temporal Information for Violent Activity Detection from Video Using Deep Spiking Neural Networks

**DOI:** 10.3390/s23094532

**Published:** 2023-05-06

**Authors:** Xiang Wang, Jie Yang, Nikola K. Kasabov

**Affiliations:** 1 Institute of Image Processing and Pattern Recognition, Shanghai Jiao Tong University, Shanghai 200400, China; wangxiang2016@sjtu.edu.cn; 2 Knowledge Engineering and Discovery Research Institute, Auckland University of Technology, Auckland 1020, New Zealand

**Keywords:** violence detection, deep learning, spiking neural network, optical flow, spatial and temporal analysis

## Abstract

Increasing violence in workplaces such as hospitals seriously challenges public safety. However, it is time- and labor-consuming to visually monitor masses of video data in real time. Therefore, automatic and timely violent activity detection from videos is vital, especially for small monitoring systems. This paper proposes a two-stream deep learning architecture for video violent activity detection named SpikeConvFlowNet. First, RGB frames and their optical flow data are used as inputs for each stream to extract the spatiotemporal features of videos. After that, the spatiotemporal features from the two streams are concatenated and fed to the classifier for the final decision. Each stream utilizes a supervised neural network consisting of multiple convolutional spiking and pooling layers. Convolutional layers are used to extract high-quality spatial features within frames, and spiking neurons can efficiently extract temporal features across frames by remembering historical information. The spiking neuron-based optical flow can strengthen the capability of extracting critical motion information. This method combines their advantages to enhance the performance and efficiency for recognizing violent actions. The experimental results on public datasets demonstrate that, compared with the latest methods, this approach greatly reduces parameters and achieves higher inference efficiency with limited accuracy loss. It is a potential solution for applications in embedded devices that provide low computing power but require fast processing speeds.

## 1. Introduction

Workplace violence, particularly in healthcare settings, has become a worrying trend worldwide [1]. For example, it was reported that during the COVID-19 pandemic more than 30% of healthcare workers and patients were exposed to violence [2]. Increasing physical workplace violence in healthcare settings has seriously challenged healthcare professionals’ health. Increasing video surveillance devices can help record violence, effectively providing clues and evidence for recognizing violence-related behaviors. It was reported that worldwide surveillance cameras generated about 3 ZB of video data in 2014, with an annual growth rate of 40% [3]. However, visually identifying violence from massive video data is both labor and time consuming. Therefore, developing methods to automatically and efficiently process big data is extremely worthwhile. Generally, video-based violence detection means recognizing violent behaviors from surveillance video data, a sub-problem of human action recognition. Video data consists of consecutive frames which represent continuous motions. Neighboring frames contain much redundant information. It is crucial to efficiently fuse spatial and temporal information in the frames for violent behavior recognition. In addition, processing the massive data quickly and in an energy-friendly manner is important, especially for embedded devices with low computing power.

Many researchers have developed methods to detect violence. Some early methods detect violence by tracking the change of relevant objects’ statistics over time instead of directly extracting the features of violent events [4]. Itsaso et al. have reviewed several methods related to video activity recognition [5]. Generally, there are two paths to detect violence in videos: the classifiers, based on feature-extraction algorithms, and the deep learning neural networks. For the first path, many classical methods commonly develop a feature descriptor or encode strategy for frames and classify the violent activity using traditional machine learning algorithms (e.g., SVM [6], AdaBoost [7]).

Many effective deep learning neural networks have recently been proposed for action recognition. C3D [8], TSN [9], and ECO [10] use convolutional neural networks (CNNs) to extract spatiotemporal features in videos. They have shown excellent performance on UCF101. FightNet is built to represent the complicated visual violence interaction by fusing features from multimodal data, including optical flow images [11]. A CNN-RNN-based network employs adjacent frame differences as the input to encode the changes occurring in videos [12]. Jain proposed a deep NeuralNet system that extracts motion features from RGB dynamic images and uses a transfer learning strategy by fine-tuning the pre-trained Inception-Resnet-V2 model [13]. Attention-based methods are also quite popular for video classification. The attention mechanism has been used in generative adversarial networks to explore the task of single image super-resolution [14]. An attention-guided convolutional neural network has been proposed for image denoising [15]. Jiang proposes a two-pathway transformer network using memory-based attention for video action recognition [16]. MM-ViT, based on a transformer, exploits all readily available modalities to identify video action [17]. Another transformer-based InspectorNet is designed to recognize violent action in animated cartoon videos, which requires significant computing resources for training [18]. Many attention-based methods for human behavior recognition use pre-training technology to reach higher accuracy on the UCF101 dataset but usually require significant computing resources. The attention-based method is not an ideal solution for small embedded devices and neuromorphic chips that require low computing power but demand fast processing speeds. Most of these existing deep learning models for detecting violence in videos include a mass of parameters. They require a lot of time and computing resources to train them. Thus, they are limited to deploying these methods in real-time scenes, such as mobile or embedded applications.

In the last decade, the spiking neural network (SNN) has achieved increasing development. It is composed of spiking neurons and mimics the human brain to process information. Compared to classical artificial neural networks, SNN shows the advantages of computational reduction and low energy costs, and is especially suited to low-power hardware platforms [19]. TDSNN converts CNNs to temporal-coding SNNs [20], which greatly reduces total operations. An improved SNN showed a median accuracy 99.5% for the discrimination of three EMG patterns [21]. S4NN achieves comparative performance with supervised multi-fully connected layers by approximating traditional error backpropagation [22]. Several gradient approximation methods have been proposed to train SNNs in a supervised manner, the so-called surrogate gradient trick [23]. A spike-based encoding method approximately encodes the activations and errors in standard feed-forward artificial neural networks to train the method using standard backpropagation [24]. Nikola Kasabov et al. proposed a spiking neuron-based architecture, namely NeuCube [25]. It has been widely explored on spatiotemporal data [26], such as EEG [27] and emotion data [28], with impressive performance. JEDMO-SNN [29] develops a descriptor for human action recognition relying on the joint entropy of difference in the optical flow feature vectors and uses a spiking neural network to aggregate the information across frames. These SNN-based methods perform well on some tasks, such as ECG classification. In many cases, real-time violence detection is significant for public security. In particular, it would help to provide surveillance in hospitals and other healthcare settings to identify and potentially reduce violence against healthcare professionals [30]. The challenge for detecting violence is exploring an effective method to extract spatiotemporal features in videos. Meanwhile, it can remarkably reduce model complexity. Thus, it is expected to be applied in small embedded devices. To the best of our knowledge, existing works have not addressed the problem well. Based on the intrinsic merit of efficiently extracting temporal features, SNN could be a promising solution.

This work proposes a spiking neuron-based deep learning model, SpikeConvFlowNet, for recognizing violent activity. A two-stream architecture is constructed using RGB frames and their optical flow data as inputs to learn the spatiotemporal features of video frames. The model is composed of multiple convolutional spiking layers. The convolutional layers are employed to extract high-level spatial features for maintaining high accuracy. Optical flow is used to strengthen the capability of extracting critical motion information. Unlike existing violence-detection methods, spiking neurons are used as the neural nodes in the two-stream model, which can effectively extract temporal features across frames by remembering historical information and improve computational efficiency. The main contribution is that by combining the advantages of optical flow, convolution, and spiking network, this model dramatically reduces parameters and achieves higher inference efficiency with limited accuracy loss.

The contents in this writing are structured as follows. Section 2 reviews some related work, covering essential backgrounds on optical flow and benchmark models. Section 3 presents the methodology and discusses the framework of SpikeConvNet and the approximate backpropagation algorithm for training the model. Section 4 shows the experimental results on four public benchmark datasets, including training details and hyperparameter configuration. The proposed method is compared with the current works in terms of computational efficiency and performance. Section 5 shows the conclusion.

## 2. Related Work

### 2.1. Optical Flow

The optical flow method is widely applied in video-based data to detect motion in two subsequent video frames using flow vectors [31]. It describes the apparent motion of brightness patterns between two successive images [32]. It insulates the moving objects from the static background objects. As a result, optical flow estimation yields a two-dimensional vector field, i.e., motion field, representing the velocities of each point of an image sequence. Optical flow can provide important information about the spatial arrangement of the objects viewed and the rate of change of this arrangement. Therefore, the methods based on optical flow can commonly achieve higher accuracy for moving object detection [33]. A local differential method is proposed, which integrates the flow constraint of one pixel into a linear system and solves each pixel’s flow independently [34]. In recent years, CNN has been applied to extract deep features of the input images, which are then integrated into common optimization algorithms to calculate optical flow [35]. This work employs the local differential method to compute optical flows based on video data. Then, spike-based CNN blocks are utilized to extract deep features of optical flows to obtain higher accuracy.

### 2.2. Benchmarks

Many neural networks for human action recognition based on different frameworks have recently been proposed. C3D [8] is a classic approach for spatiotemporal feature learning using three-dimensional deep convolutional networks. An excellent RNN-based model, Context-LSTM [36], is proposed to reduce training time and memory usage. Based on a well-pre-trained ResNet50, it constructs three LSTM layers and two fully connected layers to classify human actions. As a result, it achieves competitive detection accuracy. STS-ResNet [37] is a convolutional spiking neural network based on ResNet18. It presents a modified training method to train a new deep SNN architecture and extract features from video frames. It should be noted that optical flow technology is not used in this model to identify human actions. These three models are based on CNN, RNN, and convolutional SNN, respectively. In this work, they are used as benchmarks to evaluate our method. The proposed model outperforms or approaches the benchmarks on different datasets for violence recognition, meanwhile significantly reducing parameters (see Table 1).

## 3. Proposed Method

### 3.1. IF Neuron-Based Gradient Descent Backpropagation Algorithm

As it is known, the outputs of spiking neurons in SNNs are spike events, which are discrete over time. Therefore, the transfer function of a spiking neuron is non-differentiable, through which the gradient cannot be back-propagated [38]. Several surrogate gradient methods for spiking neurons have been proposed to handle the challenge, enabling SNNs to optimize loss using gradient descent algorithms [39]. The main idea is to adopt an approximation function to estimate the gradient of the spike generation function. For convenience, an approximation gradient descent backpropagation algorithm is briefly reviewed.

This paper uses integrate and fire (IF) as the spiking neuron model in SNN, which transmits the output signals in the form of spikes over time. An IF neuron fires a post-spike and resets the membrane potential to the initial state (zero in this work) whenever the accumulation of input currents in the membrane potential is greater than the firing threshold $V_{th}$. In an IF neuron-based SNN, this process repeats in each IF neuron over time, and input spikes are propagated and computed throughout the layers.

**Figure 1:**
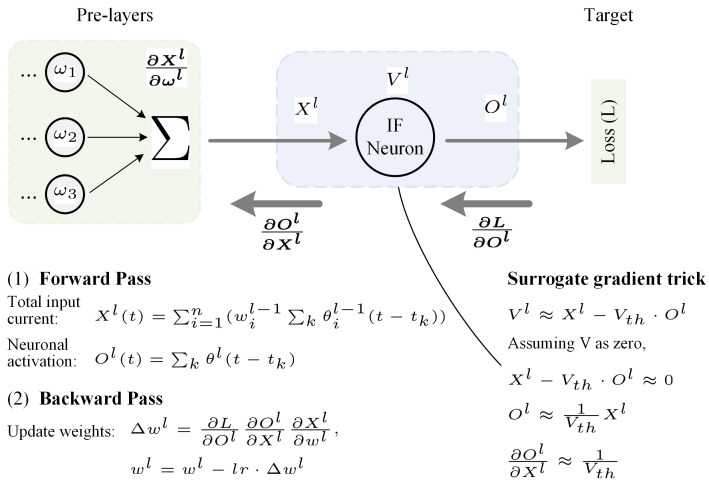
An illustration of IF neuron-based gradient BP framework. (1) Forward pass: The total input current is the weighted sum of pre-spike trains from the pre-layer, then transmitted to the next neuron.The activation of the IF neuron is sent to the post-layers to calculate the loss. (2) Backward pass: The surrogate gradient trick is used to approximately formulate the activation function of the IF neuron. The gradients of the loss function are propagated backward down to the input layer through the hidden layers using the recursive chain rule, and the weights are updated using the gradient descent algorithm.

An approximated derivative for IF neuron activation is proposed [40] to optimize loss using a gradient descent algorithm in SNN. Figure 1 illustrates the operation mechanism of an IF neuron-based gradient backpropagation (BP) algorithm. After the forward propagation, we can evaluate the loss function, usually defined as the cross-entropy between targets and actual outputs the network gives. Then, the gradients of loss function $\Delta \omega^{l}$ are propagated backward all the way down to the input layer through the hidden layers using the recursive chain rule, which can be obtained using the formula in Figure 1. $X^{l}\left(t\right)$ denotes the input current influx for a spiking neuron in the $l_{th}$ layer at time instant *t*. It equals the weighted sum of pre-spike train at each time step, and the equation is as shown in Figure 1. The $\theta_{i}\left(t-t_{k}\right)$ indicates a spike event from $i_{th}$ pre-neuron at time $t_{k}$. In this work, pre-neuron and post-neuron mean the neurons in a former layer and its following layer, respectively. At each time instant $t_{k}$, all spiking neurons in different layers can fire spikes. The activation function of an IF neuron is the sum of output spike train over time *t*, which can be formulated as $O\left(t\right)=\sum_{k}\theta \left(t-t_{k}\right)$. It describes the relationship between the weighted sum of pre-spike trains and the outputs of post-neurons over time *t*. Then, the membrane potential of the neuron in each layer can be formulated as $V\left(t\right)\approx X\left(t\right)-V_{th}\cdot O\left(t\right)$.

Due to the discontinuity of membrane potential at the time instance of firing, the spike generation function is non-differentiable. A pseudo derivative method for the activation of IF neurons in the hidden layers is used to back-propagate the output error by the chain rule. The aim is to approximately estimate $\frac{\partial O}{\partial X}$. If a hidden neuron does not fire any spike, the derivative of the corresponding neuronal activation is set to zero. The spike generation function of the IF neuron is a hard threshold function that generates the output signal as either +1 or 0. The IF neuron fires a post-spike whenever the input currents accumulated in the membrane potential exceed the firing threshold. Namely, the IF neuron generates the output signal as a binary value.

In Figure 1, $V_{th}\cdot O\left(t\right)$ accounts for the fire/reset behavior of the membrane potential. If we use the small signal approximation trick to assume *V* is zero, the activation of IF neuron $O\left(t\right)\approx \frac{1}{V_{th}}X\left(t\right)$.Then, the derivative of IF neuronal activation can be approximated as a linear function with a slope of $\frac{1}{V_{th}}$ as the straight-through estimation [41].

It should be emphasized that the spiking neuron fires a pulse when the accumulated current exceeds a threshold. Therefore, the input to a spiking neuron is the accumulation of historical inputs. In other words, the neuron can remember historical information. Therefore, it is helpful to improve computing efficiency for spiking networks.

### 3.2. SpikeConvFlowNet Architecture

It is well known that CNNs can effectively learn the high-level features of images. However, they commonly require much time and computational resources to train millions of parameters on video-based data. On the other hand, SNNs can effectively extract features for temporal data, but with the network depth increasing, spike activities significantly reduce in the case of fully fledged SNNs. This limitation is commonly called the vanishing spike phenomenon [42], which is prone to cause performance degradation in deep SNNs. Combined with CNNs, a shallow SNN-based model potentially achieves competitive performance, and could avoid the vanishing spike phenomenon.

**Figure 2:**
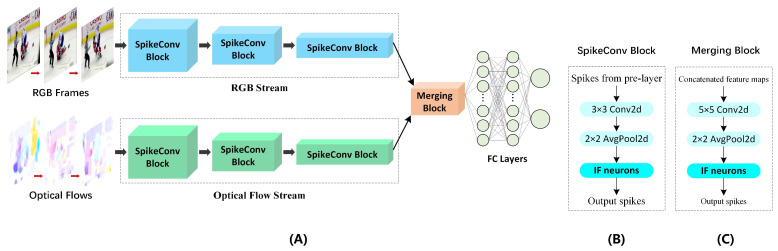
SpikeConvFlowNet architecture. (**A**) shows the topology of the method, including four parts: RGB stream, optical flow stream, merging block, and fully-connected layers; (**B**) and (**C**) show the structure of the SpikeConv Block and the merging block, respectively.

Our work proposes a deep hybrid architecture named SpikeConvFlowNet, consisting of convolution operations-based spiking neuron layers. Thus, this network can employ the benefits of SNN for processing temporal signals and CNN for maintaining performance. Figure 2 describes the structure of SpikeConvFlowNet.

**Figure 3:**
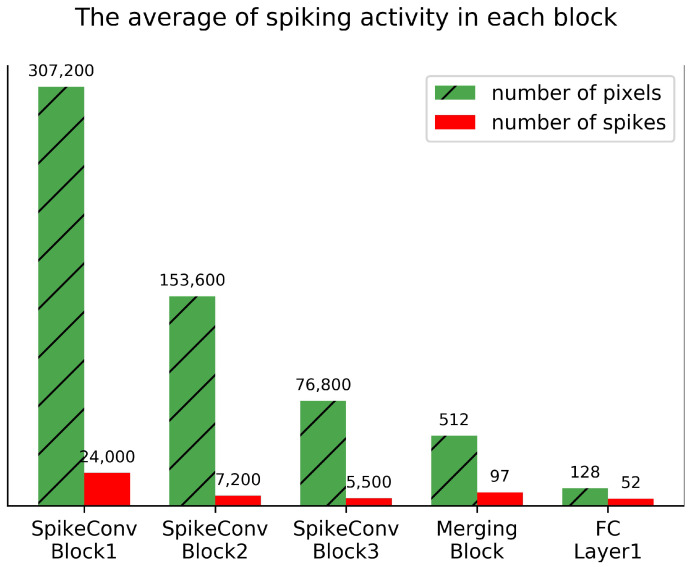
The histogram shows the average spiking activity in each block on four testing datasets. For each pair, the left denotes the number of pixels in the output of the block-layers, while the right denotes the number of spikes fired in the block-layers.

Generally, the proposed model accommodates four parts: the RGB stream, the optical flow stream, the merging block, and the fully connected layers. The RGB and optical flow streams’ inputs are RGB frames and corresponding grayscale optical flows, respectively. Optical flow can provide important information about the spatial arrangement of the objects viewed and the rate of change of this arrangement. Therefore, the methods based on optical flow would commonly achieve higher accuracy for moving object detection. Thus, combining features from RGB frames and their optical flows potentially can help us reach higher performance in our network. The RGB stream and the optical flow stream are composed of cascaded SpikeConv blocks. The SpikeConv block’s topology resembles the classical convolutional layer, which includes a 2D convolution operation followed by a 2D average pooling operation. However, a clear highlight in the SpikeConv block is using spike neurons (IF neurons in our work) for activation instead of traditional analog neurons, such as Relu or sigmoid function. Hence, the input and output for each intermediate layer in a SpikeConv block are spikes, which are binary tensors in mathematical expressions. When performing the pooling operations, the output would be quite a sparse matrix (see details in Figure 3), which could lead to much information loss if using maximum pooling. That is why average pooling is used in this paper. The images and their corresponding optical flows are consecutively processed through the RGB and optical flow streams over time. The outputs are collected in the output accumulators until the last image is finished. Then the input for the merging block in the forward phase is constructed by concatenating the feature maps from the RGB stream and the optical flow stream, which integrates one convolution layer and one pooling layer with different kernel sizes. The accumulated outputs are passed through the merging block, which can enhance the ability of feature expression by merging feature maps from two different streams. The output is flattened to vectors as the input of fully connected layers. Next, two fully connected layers are utilized to classify the type of violence. Sigmoid activation is adopted in the last layer, which enhances the ability to make the output fit the distribution of the labels and lessen the difficulty of training the model.

To sum up, this two-stream model uses the convolutional layers to extract high-level spatial features for high accuracy and uses optical flow to strengthen the capability of extracting motion information. Unlike existing CNN/RNN-based methods for violence detection, spiking neurons are used as the neural nodes for each convolutional spiking layer. The output of a spiking neuron is a binary value, which can help speed up matrix-related operations and thus improve computational efficiency. Spiking neurons can also efficiently extract temporal features across frames by remembering historical information. When training or using the model, it only needs to receive the next frame sequentially and output prediction results. Thus, this model combines the advantages of optical flow, convolution, and spiking networks.

### 3.3. Loss and Optimization

The total loss is evaluated when the network’s forward propagation of all consecutive frames is finished. This work defines the cost function as the cross-entropy between the true output and the network’s predicted distribution. To avoid the overfitting problem, we use $L_{2}$ regularization to improve the model’s generalization capability.

(1) $$Loss=\frac{1}{N}\sum_{i}^{N}\frac{1}{T}\sum_{t}^{T}\sum_{c}^{C}\left(-y_{i}^{c}\left(t\right)\cdot log\left(p_{i}^{c}\left(t\right)\right)\right)+\frac{\lambda }{2}\cdot {∥w∥}_{2}^{2}\;,$$

where *N* is the number of samples, *T* indicates the length of videos, *C* denotes the number of classes in labels, *y* is the true output, *p* denotes the network’s predicted result, *w* is parameters in the network, and λ is a hyper-parameter which represents a trade-off between the prediction error on the training dataset and the generalization capability.

An end-to-end supervised spiking training method for SpikeConvFlowNet is derived based on Section 3.1 and shown in Algorithm 1.
**Algorithm 1:** Pseudocode for training in SpikeConvFlowNet.
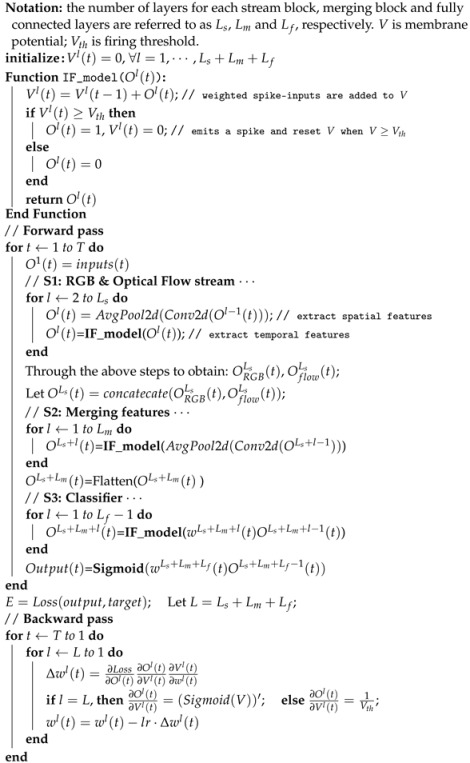


**Figure 4:**
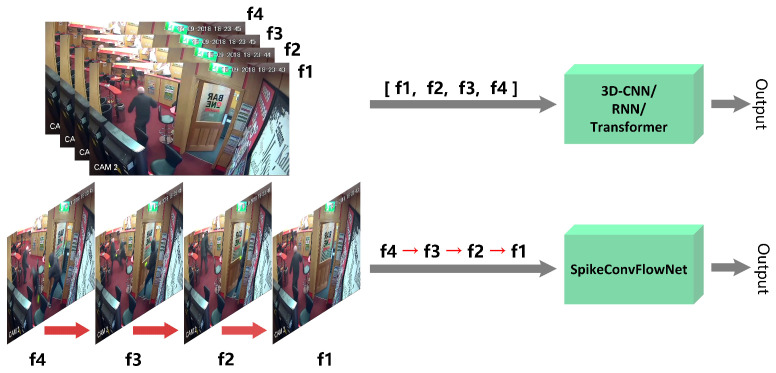
Different input paradigms between SpikeConvFlowNet and existing models. Most existing models commonly extract features from video segments, while SpikeConvFlowNet processes the frames from videos one by one, which reduces memory consumption.

In the forward pass, SpikeConvFlowNet processes the frames from video data one by one. As shown in Figure 4, each frame from the video sequentially goes through the network over time. The historical information from inputs can be recorded by firing spikes and accumulating the input signal change. For comparison, the inputs for 3D-CNN, RNN, or transformer architecture are video clips including a series of frames.Thanks to this mechanism, the model can be applied in embedded devices with less memory. The weighted spike trains from the previous layer at time *t* are integrated into the membrane potential V(t) of the IF neuron in the next layer.The neuron fires a spike when V(t) exceeds the threshold $V_{th}$, then V(t) is reset to the initial value zero. $V_{th}$ is a key hyper-parameter closely connected with firing spikes. In this work, $V_{th}$ is set as 0.75 by trial and error, and the initial membrane potential of each IF neuron is set as zero.

In particular, to achieve higher performance and reduce the difficulty of training the model, the final layer neurons integrate the weighted sum of spikes in the output accumulator without producing any spikes at the output. The sigmoid function is applied in the last layer as the activation. The error is computed by the Equation (1). In this work, Adam, a gradient descent backpropagation algorithm, is employed to optimize the loss [43]. To be specific, the gradients of the loss are propagated backward all the way down to the input layer through the hidden layers using the recursive chain rule. Meanwhile, the weights are updated using the gradient descent algorithm. The main difference between IF neuron-based SNN and the standard ANN is that the IF neuron model is discontinuous and non-differentiable. In the backward pass, sigmoid activation is used for the last layer, which is continuous and piece-wise differential. An approximate derivative for the IF neuron activation is adopted for other hidden layers, that is, using $\frac{1}{V_{th}}$ instead of $\frac{\partial O^{l}\left(t\right)}{\partial V^{l}\left(t\right)}$ in Algorithm 1.

### 3.4. Experiment Settings

This work implements all experiments using Python 3.7 and Pytorch 1.3.1. The code is run in a GPU workstation with one GeForce GTX 1080 from the Nvidia company with 128 GB memory and 16 CPUs with Intel(R) Xeon(R) CPU E5-2620 v4 @ 2.10 GHz. The operating system is Ubuntu 18.04.3.

The proposed model, SpikeConvFlowNet, is constructed as shown in Figure 2. The experiments are performed on four public violence detection datasets, namely Movies, Hockey [44], Crowd [45], and RWF-2000 [46], which can be freely available online. These datasets are from various sources. Due to this, the number of frames and their size for each video in different datasets are not uniform. Therefore, it is necessary to transform the frame size to (320, 240) by downsampling and cropping operations. To keep the information as much as possible, it is not compulsory to transform the length of the videos in each dataset to be uniform. Instead, the batch size for training the model is set as one if the video includes different lengths in the dataset, otherwise, it is set as 64. The settings of main hyper-parameters, namely threshold, learning rate, and the weight for regularization λ, are empirically set by trial and error. They are set as follows: threshold (0.75), lr (0.01), λ (0.01). Each dataset is split into three parts: the training set (70%), the validation set (10%), and the test set (20%).

## 4. Results

### 4.1. Datasets

**Figure 5:**
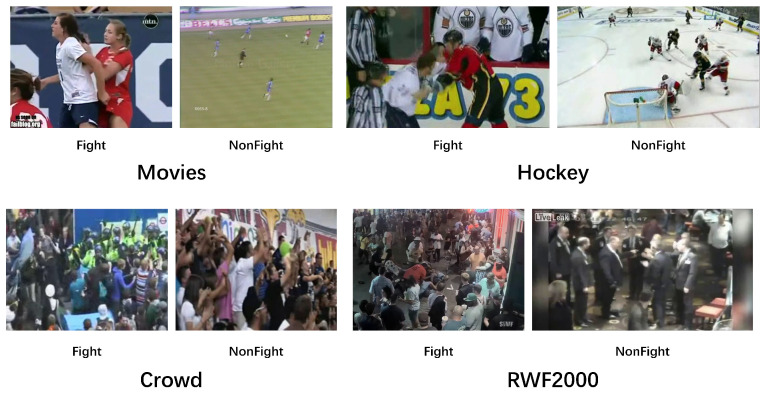
The typical scenes in four datasets. The videos in the Crowd Violence and RWF2000 datasets include more crowd and complex real-life scenes than the first two.

In this paper, four public datasets (namely Movies, Hockey, Crowd, and RWF-2000) are employed to evaluate the performance of the proposed model. They have been adopted to evaluate the capability of violence detection in many works. The first two datasets, namely the Movies Fight and the Hockey Fight, are presented by Nievas et al. The Movies Fight dataset has 200 clips extracted from short movies, while the Hockey Fight dataset has 1000 clips captured in the National Hockey League hockey games. Both of these two datasets have video-level annotations. There are 246 videos with or without violent behaviors in the Crowd Violence dataset. This dataset aims to recognize violence in crowded scenes, which includes overcrowded scenes but low image quality. The length of the videos is between 1.04 s and 6.53 s. RWF2000 dataset, collected from the YouTube platform, consists of 2000 video clips captured by surveillance cameras in real-world scenes. Most videos in this dataset include complex real-life scenes, which poses a challenge to detecting violence accurately. Figure 5 shows the typical scenes in four datasets. It is easy to find out that the scenes in the Crowd Violence and RWF2000 datasets are much more complex than the first two, which could bring more difficulties for violence detection.

### 4.2. Experimental Results

The proposed model is constructed based on the settings in Section 3.4, then trained on the four datasets, which include two types (fight and non-fight) of videos. The model can generally quickly converge into a stable value. The result shows that loss curves for the CrowdViolence and RWF2000 datasets fluctuate more than the other two. In this work, four evaluation measures, namely Accuracy, Precision, Recall and $F_{1}$, are introduced [47], and which are defined as following:

(2) $$Accuracy=\frac{TP+TN}{TP+TN+FP+FN}\;,$$

(3) $$Precision=\frac{TP}{TP+FP}\;,$$

(4) $$Recall=\frac{TP}{TP+FN}\;,$$

(5) $$F_{1}=2*\frac{Precision*Recall}{Precision+Recall}\;,$$

If a video is labeled as a fight and the model also predicts it as a fight, the predicted result is considered true positive (TP). Similarly, if a video is labeled as a non-fight and the model considers it a non-fight, the predicted result is true negative (TN). Both true positive and true negative suggest a consistent result between the predicted value and the ground truth. If a video with the fight label is predicted as a non-fight by the model, the prediction is false positive (FP). Similarly, if a video with the non-fight label is suggested as a fight by the model, the prediction is false negative (FN).

To evaluate the performance, we compared the proposed model with seven existing models, which are 3D ConvNet [48], ConvLSTM [12], C3D [8], I3D(Fusion) [49], Flow Gated Network [46], Context-LSTM, and STS-ResNet [37], respectively. Table 2 shows predicting accuracy on testing data and the number of parameters based on SpikeConvFlowNet and other methods. The performances based on testing data of the first five models (from 3D ConvNet to flow gated network) are reported in the papers. It should be noted that the methods labeled using an asterisk in Table 2 are implemented and trained by ourselves to obtain their test accuracy values.

The mean and variance of the training accuracy for the other seven models are not reported in their articles. To verify the stability of the proposed model, we trained and evaluated it five times. The training data and testing data are randomly split for each experiment. The mean and variance of the testing accuracy are as follows: Movies (mean: 100%, variance: 0.00), Hockey (mean: 98.39%, variance: 0.26%), Crowd (mean: 90.14%, variance: 1.83%), RWF2000 (mean: 88.35%, variance: 1.77%).

To further test the performance of the proposed method on datasets with multiple classes, two public human action recognition datasets are used, namely HMDB51 and UCF101 [29]. The HMDB51 dataset consists of 6766 video classes annotated for 51 action classes extracted from movies and YouTube. The UCF101 dataset contains 13,320 video clips categorized into 101 action classes collected from YouTube. Our model is compared with two methods for recognizing human actions: Context-LSTM and STS-ResNet. All predicted results on testing data for each method are shown in Table 3. It is noted that Context-LSTM is implemented by ourselves and then the testing accuracy on HMDB51 is obtained, which is labeled by an asterisk in the table. Other results are reported in their articles.

The results in Table 2 reveal that the methods are over-fitted on the Movies Fight dataset. The main reason is that this dataset contains 200 clips, with 100 positive and negative samples each. It is balanced, but its size is small, and the scenes in clips are relatively simple. For example, fierce fighting between two or more people is considered violent, while walking or playing football is considered non-violent. As a result, it is easier to identify violent actions on the Movies dataset than on other datasets containing many complex real-life scenes. Hence, we can conclude that this dataset is no longer suited to evaluate the deep learning-based models for violence detection effectively. The performances on the Crowd Violence and RWF2000 datasets have not achieved as high testing accuracy as the first two datasets. The main reason is that these two datasets consist of many real-world scenes, such as crowd activities, which enhances the difficulty of recognizing fight or non-fight violence from these scenes. Among these methods, Context-LSTM is based on RNN and performs best. One reason is that it employs a ResNet50 well-pre-trained on large-scale datasets. It is not an ideal model for small embedded devices and neuromorphic chips. Compared to STS-ResNet, the proposed model achieves comparative performance and only includes around one percentage of parameters. A possible reason is that optical flow data enhances our method’s capability of extracting critical motion information. Based on Table 3, it can be seen that the performance of two SNN-based models drops significantly on large-scale datasets with multi-classes for action recognition. The main reason is that the HMDB51 and UCF101 datasets contain multiple complex scenes. Currently, these two convolutional spiking neural networks do not have enough ability to learn the features and recognize multi-classes actions effectively over these challenging datasets. Generally, compared with existing CNN/RNN-based methods and convolutional SNN, SpikeConvFlowNet can achieve comparative performance across all datasets related to violence detection, but also dramatically reduces the number of parameters. Moreover, the results also verify that the shallow SNN-based model can perform well while avoiding the vanishing spike phenomenon.

The training time and inference efficiency are experimentally measured to verify the proposed model’s computational efficiency further. All experiments are implemented on RWF2000, including more violent videos and complex scenes than the other three datasets. The configuration of hardware is described in Section 3.4. As shown in Table 2, Context-LSTM (RNN-based) and STS-ResNet (SNN-based) are selected as benchmarks to compare with the proposed method. To speed up the training process, all videos are pre-processed and transformed to the form of ndarray, which is a data type in Numpy packages. These models are generally convergent after being iteratively trained for 100 epochs. The GPU server is used to train these models. However, the inference efficiency is measured only using the CPU, similar to the devices with low computational resources. The training efficiency is measured by running time with the unit of the hour, while the inference efficiency is measured by frames per second (fps). The training time and inference efficiency are shown in Table 4.

The results demonstrate that the proposed model can achieve higher training and inference speed than the benchmarks. It is noted that the batch size for training is set as one due to the inconsistency of video length, which makes training time increase. Compared with Context-LSTM, the proposed model only includes one-tenth of the parameters. However, their training time is close. One reason is that SpikeConvFlowNet employs optical flow, which would slow down the training process.

Based on the results of Table 2, Table 3 and Table 4, it can be concluded that the proposed method is more efficient than existing CNN/RNN-based models and is more suitable for low-power embedded devices. Compared with the best convolutional spiking neural network, this method significantly reduces the number of parameters with limited accuracy loss. It shows that our method can provide a potential solution for neuromorphic chips to detect violent behaviors efficiently.

**Figure 6:**
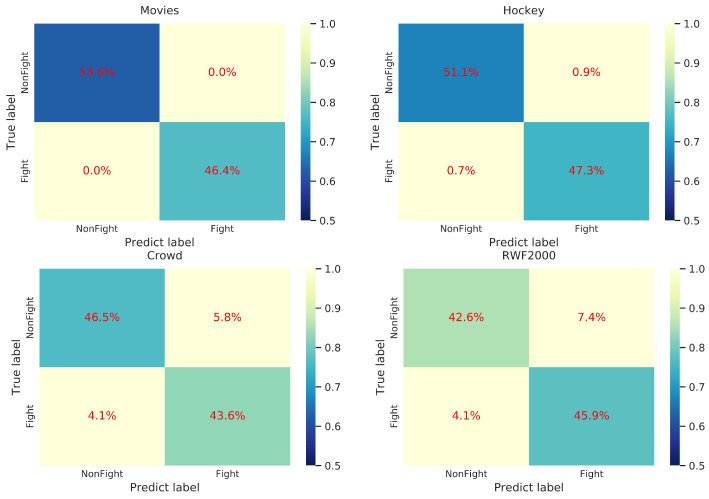
Confusion matrix of experimental results on four datasets using SpikeConvFlowNet.

Figure 6 shows the confusion matrix of experimental results on four datasets using the proposed model.

It also demonstrates that the videos with complex scenes in the last two datasets could degrade the prediction accuracy compared with other datasets. Based on the confusion matrix, Precision and Recall are calculated, shown in Table 5.

Generally, the Recall is relatively higher than the Precision. It reveals that the proposed model has a higher possibility of classifying a video as a fighting type. It can avoid omitting fight samples in testing data, but part non-fight videos could be falsely predicted as fight videos, which is acceptable for practical applications.

Furthermore, to experimentally explore the mechanisms of the SpikeConvFlowNet, Figure 3 shows the average of spiking activity in each block on four testing datasets.

**Figure 7:**
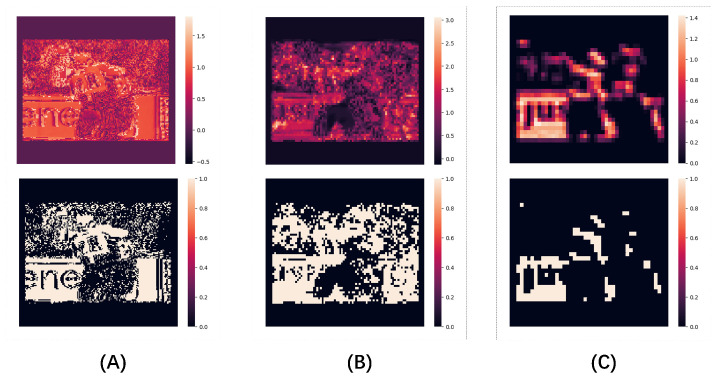
Samples from the feature maps in hidden layers. (**A**–**C**) denote SpikeConvBlock1, SpikeConvBlock2, and SpikeConvBlock3 in the RGB stream. As for each heatmap pair, the above is before operating through the IF neuron, while the below is for the spiking activity after the IF neuron. It shows that the output of each layer is a sparse matrix.

It reveals that the spikes number generally accounts for quite a low percentage (less than 10% in the first three SpikeConvBlocks) in the output of each block layer. In other words, the output for each block is a relatively sparse binary tensor. Figure 7 shows several samples from the feature maps in hidden layers, i.e., SpikeConvBlock1, SpikeConvBlock2, and SpikeConvBlock3.

For each layer, the heatmaps before operating through the IF neuron and after the IF neuron are displayed. It could be inferred that the proposed model is capable of extracting spatial features by spiking neurons. These heatmaps also visually show the sparsity of spikes fired in each layer. This character enables the proposed architecture to be energy-efficient for the hardware solutions of SNNs.

To sum up, the spiking neuron fires a pulse when the accumulated current exceeds a threshold. Therefore, the input to a spiking neuron is the accumulation of historical inputs. In other words, the neuron can remember historical information. Therefore, when training or using the model, it only needs to receive the next frame sequentially and outputs prediction results. CNN-based models need many historical frames to output results, which consumes more computing time and hardware resources. However, it is worth noting that LSTM-based models can also remember historical information similar to spiking neurons to output prediction results. Moreover, the output of each spiking neuron is a binary value. Many matrix multiplication operations are involved in training and using deep neural networks. The binary matrix can significantly improve matrix multiplication operations’ computational efficiency, saving time and computing resources. CNN/RNN-based models do not have this advantage. Compared with existing CNN/RNN-based models and convolutional SNN, this method can improve computing efficiency and save computing time and hardware resources, which is verified by the experimental results. Meanwhile, it dramatically reduces parameters and achieves higher inference efficiency with limited accuracy loss by combining the advantages of optical flow and convolutional spiking networks. In addition, since the output of spiking neurons is a discontinuous spike train, the combination of SNN and neuromorphic chips can further improve computational efficiency and reduce power consumption.

## 5. Conclusions

In this paper, we propose a supervised deep learning spiking neural network (SpikeConvFlowNet) for video data-based violence detection, which is composed of multiple convolutional spiking layers.The convolutional layers are used to extract high-level spatial features for high accuracy, while spiking neurons are employed to extract temporal features effectively. An optical flow-based method is utilized to capture motion features to enhance the capability of recognizing violence. An IF neuron-based algorithm is derived to optimize a loss function with the regularization term. The model is trained and evaluated on public datasets. The experimental results show that the proposed method for violence detection achieves a comparative performance and dramatically reduces the number of parameters compared with all benchmark methods. More tests demonstrate that this model can decrease training time and improve inference efficiency. The sparsity of spiking activity potentially makes the model energy efficient for hardware solutions. Furthermore, sequentially processing the images in this architecture would conserve memory consumption and be helpful for online applications and embedded devices that require low computing power but demand fast processing speed. 

## Figures and Tables

**Table 1:** SpikeConvFlowNet compared to benchmarks’ results. The model dramatically reduces parameters with limited accuracy loss.

Method	Hockey	Crowd	RWF	Params (M)
C3D	96.5%	84.4%	82.8%	94.8
Context-LSTM	99.2%	93.8%	92.3%	1.7
STS-ResNet	98.9%	91.2%	88.3%	11.7
SpikeConvFlowNet	98.4%	90.2%	88.5%	0.178

**Table 2:** Experimental accuracy on four datasets and the number of parameters in these models. The asterisk represents that the methods are implemented by ourselves, and other results in the table are cited from existing works.

Method	Movies	Hockey	Crowd	RWF	Params (M)
3D ConvNet	99.97%	99.6%	94.3%	81.7% *	86.9
ConvLSTM	100%	97.1%	**94.5%**	77.0%	47.4
C3D	100%	96.5%	84.4%	82.8%	94.8
I3D (Fusion)	100%	97.5%	88.9%	81.5%	24.6
Flow Gated Network	100%	98.0%	88.8%	87.2%	0.27
Context-LSTM *	100%	99.2%	93.8%	**92.3%**	1.7
STS-ResNet *	100%	98.9%	91.2%	88.3%	11.7
SpikeConvFlowNet	100%	98.4%	90.2%	88.5%	**0.178**

**Table 3:** The comparison of predicting accuracy for multi-classes task on HMDB51 and UCF101. The asterisk represents that the result is obtained by ourselves, and other results are published in existing works.

Method	HMDB51	UCF101
Context-LSTM	80.1% *	92.2%
STS-ResNet	21.5%	42.1%
SpikeConvFlowNet	23.6%	40.7%

**Table 4:** Training time and inference efficiency on RWF2000.

Method	Training Time(h)	Inference Efficiency(fps)
Context-LSTM	2.4	290.7
STS-ResNet	5.1	170.3
SpikeConvFlowNet	2.2	372.8

**Table 5:** Precision and Recall for SpikeConvFlowNet on four datasets.

Measures	Movies	Hockey	Crowd	RWF
Precision	1.00	0.98	0.88	0.86
Recall	1.00	0.99	0.91	0.92
$F_{1}$	1.00	0.99	0.90	0.89

## Data Availability

The datasets used in this work can be accessed on the following link: https://drive.google.com/drive/folders/1DUpRySTKNTot-_eLHn8lRxhlbmGhVLL6q?usp=sharing (accessed on 22 February 2023).
